# Obstetric care providers’ knowledge, practice and associated factors towards active management of third stage of labor in Sidama Zone, South Ethiopia

**DOI:** 10.1186/s12884-017-1480-8

**Published:** 2017-09-07

**Authors:** Zelalem Tenaw, Zemenu Yohannes, Abdela Amano

**Affiliations:** 1 School of Nursing and Midwifery, College of Medicine and health sciences, Hawassa University, Hawassa, Ethiopia; 2 School of Public and Environmental Health, College of Medicine and health sciences, Hawassa University, Hawassa, Ethiopia

**Keywords:** Active management of third stage of labor, Ethiopia, Knowledge, Practice, Third stage

## Abstract

**Background:**

Active management of third stage of labor played a great role to prevent child birth related hemorrhage. However, maternal morbidity and mortality related to hemorrhage is high due to lack of knowledge and skill of obstetric care providers ‘on active management of third stage of labor.

Our study was aimed to assess knowledge, practice and associated factors of obstetric care providers (Midwives, Nurses and Health officers) on active management of third stage of labor in Sidama Zone, South Ethiopia.

**Methods:**

An institution based cross sectional study design was conducted from December 1–30 /2015 among midwives, nurses and health officers. Simple random sampling technique was used to get the total of 528 participants. Data entry was done using EPI Info 3.5.1 and exported to SPSS version 20.0 software package for analysis. The presence of association between independent and dependent variables was assessed using odds ratio with 97% confidence interval by applying logistic regression model.

**Results:**

Of the 528 obstetric care providers 37.7% and 32.8% were knowledgeable and skilled to manage third stage of labor respectively. After controlling for possible confounding factors, the result showed that pre/in service training, being midwife and graduation year were found to be the major predictors of proper active management of third stage of labor.

**Conclusion:**

The knowledge and practice of obstetric care providers towards active management of third stage of labor can be improved with appropriate interventions like in-service trainings. This study also clearly showed that the level of knowledge and practice of obstetric care providers to wards active management of third stage of labor needs immediate attention of Universities and health science colleges better to revise their obstetrics course contents, health institutions and zonal health bureau should arrange trainings for their obstetrics care providers to enhance skill.

## Background

Active management of third stage of labor played a great role to prevent childbirth related hemorrhage. Proper practice of active management of third stage of labor is a novel method to alleviate postpartum hemorrhage [1, 2].

Hemorrhage is the leading cause of maternal death, especially in developing countries including Ethiopia. Maternal mortality ratio in Ethiopia per 100,000 live births were 676 in 2012 and 420 in 2014. A 2015 estimate puts the maternal mortality ratio in Ethiopia 353 /100,000.

Worldwide, maternal morbidity and mortality is alarmingly decrease, however in developing countries, especially sub-Saharan Africa frontline maternal death is caused by hemorrhage due to infrastructure limitation, lack of skill birth attendants, inappropriate management of active third stage of labor, [2].

Third stage of labor is the period after the fetus is delivered until the placenta completely removed. It is the easiest and shortest time, but dangerous as most maternal deaths were occurred [3].

Active management of third stage of labor involves the obstetric care providers to carry out three interrelated but independent processes: - Prophylactic administration of an uterotonic agent, Controlled cord traction and uterine massage. Active management of third stage of labor is an interventions needed to reduce maternal death due to PPH [4].

FIGO–ICM Recommends to use uterotonic drugs immediately following delivery of the fetus, controlled cord traction and uterine massage immediately after delivery of the placenta, followed by massage of the uterus every 15 min for 2 h to assess the continued need for massage [5].

Active management of third stage of labor is a proven solution to prevent unnecessary procedures and complications, such as manual removal of the placenta and postpartum hemorrhage [6].

Since all parturient women are at risk for PPH, obstetric care providers need to possess the necessary knowledge and skills of active management of the third stage of labor properly to prevent PPH [7].

The WHO technical update, assures that now a days the most effective approach to prevent PPH is active management of the third stage of labor (AMTSL) [8].

Effective use of AMTSL in reducing PPH and the need for PPH treatment has been investigated by a number of large trials. The Hinchinbrook 12 randomized control trials provided evidence that AMTSL significantly reduces postpartum hemorrhage, decreases blood loss and decreases the need for blood transfusions [2].

The AMTSL practice of obstetric care providers in developing countries is not in line with what is recommended by FIGO because of certain factors like knowledge, qualification, training, and other demographic factors. The practice of AMTSL according to the FIGO/ICM recommendations in Ethiopia was only 5% of all observed deliveries [5].

The aimed of this study is to assess knowledge, practice and associated factors of obstetric care providers (amongst midwives, nurses and health officers) on active management of third stage of labor in Sidama Zone, South Ethiopia.

## Methods

An Institution based cross-sectional study was conducted among obstetric care providers in Sidama Zone from December 1–30, 2015. Sidama zone is one of the zone found in southern nation’s nationalities and peoples region (SNNPR) of Ethiopia.

According to Sidama zone health department, the total population in 2014/2015 is expected to be 3,676,576. The health institutions which are found in the zone include three governmental hospitals, 130 governmental health centers, 524 posts. Regarding human resource for health, the zone has 1857 obstetrics care providers.

Out of 19 Woredas (districts), from the Zone seven were selected by simple random sampling techniques [9]. The study population was randomly selected obstetric care providers. The sample size was determined using single population proportion formula at 95% of confidence interval with assumption of prevalence of AMTSL practice in Ethiopia 5% [5] with (**α = 0.05), 3%** marginal error **(d = 0.03).**

Multistage stage sampling method was employed by using design effect of 2 and 10% non-response response. The final sample size was 528 obstetric care providers. To collect the data, initially all public health institution in Sidama zone from selected Districts were listed and identified. The participants were allocated proportionally to each public health institution and were selected by using simple random sampling technique from each public health institution. Obstetric care providers who had service greater than 6 month were participated in the study.

The structured interviewer administered questionnaires were included sociodemographic characteristics, personal characteristics and knowledge while observational checklist for skill part assessment were used as data collection instruments.

Obstetric care provider who knew all AMTSL components, right time of oxytocin administration and cord clamping were considered as knowledgeable and the obstetric care provider who administered oxytocin with in 1 min, apply CCT and perform uterine massage considered as skilled.

Pretest was done on 5% obstetric care providers working out of the selected health to check clarity, length and completeness of the questionnaires and observation check list. Based on this necessary correction was done accordingly.

Data was collected by face to face interview using a structured and pre-tested questionnaire to assess knowledge and observation check lists for practice assessment.

Both interview and observation were used for the same participant, interview was administered to assess the sociodemographic characteristics and knowledge of the participant. After interview, verbal consent was obtained from the parturient mothers, and the participant was observed while managing third stage of labor.

Interview and observation were performed by obstetric care provider data collectors. Both sexes were participated in data collection. Seven (07) obstetric care providers who have BEmONC training were recruited and training was given for 01 days on the objective, relevance of the study, confidentiality of information, respondent rights, informed consent, and technique of interview, 02 Health professional who have 1st degree (BSC nurse, midwife or HO) were trained and supervise the data collection. Data entry was done by using EPI Info 3.5.1 and exported to SPSS version 20.0 software package for analysis. The presence of association between independent and dependent variables was assessed using odds ratio with 97% confidence interval by applying logistic regression model.

Ethical clearance was obtained from College of Medicine and Health Sciences ethical review committee, Hawassa University. Formal letter of cooperation was written for Sidama Zone Health Department and Sidama zone selected District Health Offices. After informing the objective of the study, consent was obtained voluntarily from each study subject.

## Results

### Socio-demographic characteristic and experiences of obstetric care providers

A total of 528 obstetric care providers were participated in the study, with 96.4% response rate. Out of the total respondents, 75.4% (*n* = 398) were females and the age of participants were from 22 to 45 years old. The mean age of the study population was 26.4 with SD 3.05 years. Sidama was a dominant ethnic group, which accounted for 49.6% (*n* = 262) (Table 1).

**Table 1:** Socio-demographic characteristics of the obstetric care providers

Variables		Frequency (*n* = 528)	Percentage
Sex	Male	130	24.6
	Female	398	75.4
Age	20–30	461	87.3
	31–40	57	10.8
	41–50	10	1.9
Marital status	Single	258	48.9
	Married	249	47.2
	Divorced	16	3
	Widowed	5	0.9
Ethnicity	Sidama	262	49.6
	Amhara	104	19.7
	Oromo	125	23.7
	Tigre	20	3.8
	Others®	17	3.2
Religion	Protestant	216	40.9
	Orthodox	233	44.1
	Muslim	38	7.2
	Catholic	41	7.8
Work place	Health center	483	91.5
	Hospital	45	8.5
Profession	Health officer	30	5.7
	BSc midwife	66	12.5
	Diploma midwife	330	62.5
	BSc Nurse	40	7.6
	Diploma Nurse	61	11.6
	Others➲	1	0.2
AMTSL related In/pre service training	Yes	374	70.8
	No	154	29.2
Conduciveness of delivery room	Yes	467	88.4
	No	61	11.6
Adequate oxytocic drugs	Yes	488	92.4
	No	40	7.6

Others®: Wolayta, Hadya, Kembata

Others➲ Public Nurse

### Knowledge of obstetrics care providers on active management of third stage of labor

The knowledge of the obstetrics care providers towards active management of third stage of labor were 37.7%(*n* = 199) (Table 2).

**Table 2:** Knowledge of the obstetric care providers on active management of third stage of labor

Variables		Frequency	Percent
Uterotonic drugs know	Oxytocin	439	83
	Ergometrine	57	10.8
	Misoprostol	18	3.4
	All	15	2.8
Dose of oxytocin know	0.5 mg	6	1.0
	10 IU	492	93.2
	10 mg	26	4.9
	0.5 IU	5	0.9
Recommended rout of oxytocin know	IV	34	6.4
	IM	494	93.6
Time of uterotonic drug administration know	After the delivery of anterior shoulder	33	6.2
	Within one minute after delivery of baby	451	85.3
	Within three minutes	45	8.5
Mentioned essential components of active management of third stage of labor	Administer uterotonic drugs	61	11.5
	Apply counter cord traction	102	19.3
	Uterine massage	59	11.2
	All	307	58
	Knowledgeable	199	37.7
Knowledge	Not knowledgeable	329	62.3

### Practice of obstetrics care providers on active managements of third stage of labor

The practice of the obstetrics care providers towards active management of third stage of labor were 32.8%(*n* = 173) (Table 3).

**Table 3:** Practices of the obstetric care providers on active management of third stage of labor

Variables		Frequency	Percent
Abdomen palpated to rule out the presence of second baby	Yes	331	62.7
	No	197	37.3
Uterotonic drugs given	Oxytocin	432	81.8
	Ergometrine	61	11.6
	Misoprostol	30	5.7
	Not given	5	0.9
Dose of uterotonic drugs given	0.5 mg	24	4.5
	10 IU	486	96.6
	0.5 mg	12	2.3
	Others	6	1.1
Route of uterotonic drugs given	IM	519	97.2
	IV	9	1.7
	Oral	6	1.1
Wait uterine contraction 2–3 min to apply CCT	Yes	243	46
	No	285	54
Wait gush of blood	Yes	283	53.6
	No	245	46.4
Counter cord traction applied	Yes	472	89.4
	No	56	10.6
Placenta supported by two hands during placenta delivery	Yes	421	79.7
	No	107	20.3
Membrane extracted gently with lateral movement	Yes	293	55.5
	No	235	44.5
Uterine massage immediately after delivery of placenta	Yes	227	43
	No	301	57
Uterine relaxation ensured	Yes	161	30.5
	No	367	69.5
Inform and demonstrate the mother massage uterus	Yes	196	37.1
	No	332	62.9
Skill	Skilled	173	32.8
	Not skilled	355	67.2

### Factors associated with obstetric care provider’s knowledge to wards AMTSL

Profession and year of graduation were factors which associate with knowledge of obstetric care provider’s towards active management of third stage of labor (Table 4).

**Table 4:** Factors associated with obstetrics care providers’ knowledge on active third stage management of labor

Characteristics		AMTSL knowledge		OR(97% CI)		*P* value
		Yes	No	Crude	Adjusted	
Sex	Female	154	244	1.2 (0.75–1.88)	0.86(0.54–1.34)	0.47
	Male	45	85	1.00		
Age	20–30	171	289	1.14 (0.64–2.04)	0.90 (0.50–1.62)	0.70
	>30	28	40	1.00		
Marital status	Single	114	144	1.72 (0.65–1.42)	0.92 (0.61–1.40)	0.66
	Married	85	185	1.00		
Work place	Hospital	22	23	0.55 (0.28–1.08)	0.60 (0.30–1.24)	0.13
	Health center	307	176	1.00		
Profession	Midwife	259	167	1.37 (1.07–2.60)*	**1.76 (0.33–0.87)** ^**a**^	**0.007**
	Others Θ	70	62	1.00		
Year of graduation	2005–2007	136	193	1.55 (1.00–2.29)*	**0.67 (0.46–0.98)** ^**a**^	**0.036**
	Before 2005	62	136	1.00		
Year of experience	½-2 years	105	159	1.19 (0.80–1.76)	0.84 (0.56–1.27)	0.36
	>2 years	94	170	1.00		
Receiving training	Yes	59	95	1.04 (0.63–1.48)	0.86(0.46–1.58)	0.58
	No	140	234	1.00		
Trained topic related to AMTSL	Yes	96	151	1.10 (0.75–1.62)	0.99 (0.56–1.76)	0.96
	No	103	176	1.00		

*P-value <0.05,

^a^ Adjusted for socio demographic characteristics and some concepts of AMTSL

Others Θ: Health officers, BSc/diploma nurses, public nurse

### Factors associated with obstetric care provider’s practice to wards AMTSL

Pre/in service training was associated with the practice of obstetric care providers to wards active management of third stage of labor (Table 5).

**Table 5:** Factors associated with obstetrics care providers’ practices on active third stage management of labor

Characteristics		AMTSL Practice		OR (97% CI)		*P*-value
		Yes	No	Crude	Adjusted	
Sex	Male	45	85	1.12 (0.70–1.78)	1.30 (0.78–2.16)	0.27
	Female	128	270	1.00	1.00	
Age	20–30	157	303	1.68 (0.29–1.09)	1.75 (0.89–3.43)	0.07
	>30	16	52	1.00	1.00	
Marital status	Single	108	191	1.43 (0.46–1.06)	0.73 (0.48–1.11)	0.10
	Married	65	164	1.00		
Profession	Midwives	125	271	0.81 (0.78–1.96)	0.73 (0.45–1.18)	0.15
	Others	48	84	1.00	1.00	
Conduciveness of delivery room	Yes	147	320	0.62 (0.34–1.13)	0.66 (0.35–1.25)	0.15
	No	26	35	1.00	1.00	
Place of work	Hospital	20	25	1.73 (0.29–1.15)	0.55 (0.27–1.10)	0.06
	Health center	153	330	1.00	1.00	
Year of graduation	2005–2007	102	227	0.81 (0.54–1.22)	1.28 (0.84–1.95)	0.21
	Before 2005	71	128	1.00	1.00	
Experience Year	½-2 year	87	177	1.02 (0.68–1.41)	0.97 (0.67–1.42)	0.91
	>2 year	86	178	1.00	1.00	
Receiving training	Yes	143	231	2.56 (1.56–4.20)*	**2.67 (1.60–4.50)** ^a^	**000**
	No	30	124	1.00	1.00	
Training topic related to AMTSL	Yes	98	183	1.23 (0.82–1.84)	1.28 (0.84–1.95	0.20
	No	75	172	1.00	1.00	

*P-value <0.05,

^a^Adjusted for socio demographic characteristics and some concepts of AMTSL

**Fig. 1:**
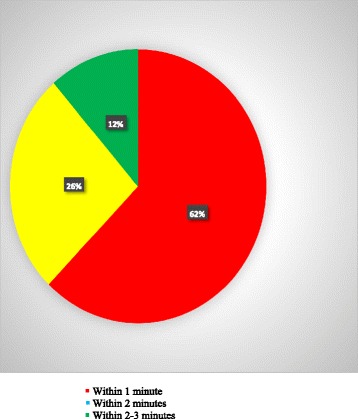
Practice of cord clamping time of obstetrics care providers (*N* = 528)

In this study 11.4% (*n* = 60) of the obstetric care providers were clamp the cord within the recommended time which is within 2–3 min (Fig. 1).

**Fig. 2:**
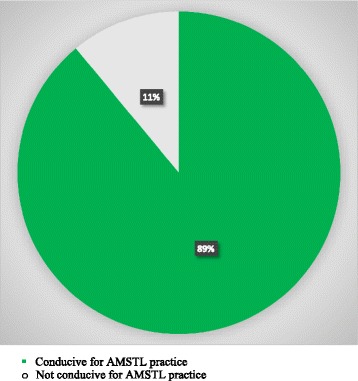
Conduciveness of delivery room

Eighty eight point 4 % (*n* = 467) of the delivery rooms were conducive to apply active third stage management (Fig. 2).

## Discussion

The available reports and this study showed that in Ethiopia the knowledge and practice of obstetric care providers towards active management of third stage of labor is unsatisfactory. Among the participants 37.7% (*n* = 199) of the obstetric care providers were knowledgeable on managing of third stage of labor actively. This finding is higher than the study conducted in south Nigeria and Tanzania 28.3% and 9% respectively [10, 11]. Profession and year of graduation were the factors which associate with obstetric care provider’s knowledge towards active management of third stage of labor. Even observational studies are exposed to observational bias, utilization of both structured interviewer administered questionaries and observation check list is considered as strength of this study. This study is the first of its kind in southern Ethiopia which includes observational check list to assess the actual practice of obstetric care providers towards active management of third stage of labor. Based on the observation the practice of obstetric care providers were not satisfactory in this study even it is better from the previous findings in Ethiopia and Nigeria [5, 9]. Almost all the obstetric care providers were rid of the placenta after administration of uterotonic drugs, like that of Australia, Holland and United Kingdom practice, but different from some United States and Canada which advocates withholding uterotonic administration until the placenta is delivered [12]. All obstetric acre providers were used oxytocin as an uterotonic drug for AMTSL which is slightly different from a study conducted in Istanbul, Turkey [6]. Most of the obstetric care providers check presence of second twine before administration of oxytocin which is better than Istanbul Turkey practice [6]. Majority of the participants were observed while correctly apply counter cord traction practice but half of them were not wait uterine contraction like that of Nepal [13] Practice. Participants who got pre/in service training were observed while correctly practicing AMTSL than who did not have training which indicates AMTSL related training is needed.

In this study majority of the obstetric care providers were midwives which is totally different from a study conducted in Ethiopia, which concludes nurses performed most (61%) in Ethiopia [5]. Physicians were not observed during active management of third stage of labor, this might be due to Physicians tend to manage more complicated third stages**.** Most of our participants were not clamp the cord with the recommended time which is within 2–3 min. In Albanian maternity hospital the practice is within 20 s [14]. There was no problem on delivery room conduciveness and availanlity of oxytocic drugs to practice AMTSL in our study area.

## Conclusion and recommendation

The knowledge and practice of obstetric care providers towards active management of third stage of labor can be improved with appropriate interventions like in-service trainings. This study also clearly showed that the knowledge and practice of obstetric care providers to wards AMTSL which needs immediate attention of Universities and health science colleges better to revise their obstetrics course contents, health institutions and zonal health office need to arrange trainings for their obstetrics care providers to enhance skill.

## Abbreviation

AMTSL: Active management of third stage of labor
AOR: Adjusted odds ratio
BEmONC: Basic emergency obstetrics and newborn care
E.C: E Ethiopian calendar
FIGO: Federation of international gynecology and obstetrics
ICM: International cooperation of midwifery
PPH: Postpartum hemorrhage
SNNPRS: Southern nation nationalities people regional state
WHO: World health organization
